# Vision Defense: Efficient Antibacterial AIEgens Induced Early Immune Response for Bacterial Endophthalmitis

**DOI:** 10.1002/advs.202202485

**Published:** 2022-07-06

**Authors:** Tingting Li, Yan Wu, Wenting Cai, Dong Wang, Chengda Ren, Tianyi Shen, Donghui Yu, Sujing Qiang, Chengyu Hu, Zheng Zhao, Jing Yu, Chen Peng, Ben Zhong Tang

**Affiliations:** ^1^ Department of Ophthalmology Shanghai Tenth People's Hospital School of Medicine Tongji University Shanghai 200072 China; ^2^ Center for AIE Research Shenzhen Key Laboratory of Polymer Science and Technology Guangdong Research Center for Interfacial Engineering of Functional Materials College of Materials Science and Engineering Shenzhen University Shenzhen 518060 China; ^3^ Shenzhen Institute of Molecular Aggregate Science and Engineering School of Science and Engineering The Chinese University of Hong Kong Shenzhen 518172 China; ^4^ Department of Radiology Shanghai Public Health Clinical Center Fudan University Shanghai 201508 China

**Keywords:** aggregation‐induced emission, endophthalmitis, immune response, photodynamic antibacteria, visual function protection

## Abstract

Bacterial endophthalmitis (BE) is an acute eye infection and potentially irreversible blinding ocular disease. The empirical intravitreous injection of antibiotic is the primary treatment once diagnosed as BE. However, the overuse of antibiotic contributes to the drug resistance of pathogens and the retinal toxicity of antibiotic limits its application in clinic. Herein, a cationic aggregation‐induced emission luminogens named with triphenylamine thiophen pyridinium (TTPy) is reported for photodynamic treatment of BE. TTPy can selectively discriminate and kill bacteria efficiently over normal ocular cells. More importantly, TTPy shows excellent antibacterial ability in BE rat models infected by *Staphylococcus aureus*. Meanwhile, the bacterial killing behavior triggered by TTPy induces innate immune response at an early stage of infection, limiting subsequent robust inflammation and protecting retina from bacterial toxins and inflammation‐induced bystander damage. In addition, TTPy performs better antibacterial ability than commercially used Rose Bengal, suggesting its excellent capability of vision salvage in acute BE. This study exhibits an efficient photodynamic antibacterial treatment to BE, which induces an early intraocular immune response and saves useful vision, endowing TTPy a promising potential for clinical application of ocular infections.

## Graphical abstract

A cationic aggregation‐induced emission luminogen (TTPy) to treat bacterial endophthalmitis (BE), which can selectively kill bacteria without obvious cytotoxicity, is presented. TTPy exhibits outstanding antibacterial ability and alleviates the inflammation in BE due to its induced innate immune response. Moreover, compared to Rose Bengal, TTPy protects the retina and visual function, providing a new strategy for treating BE.

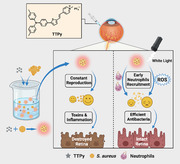

## 1 Introduction

Bacterial endophthalmitis (BE) is a devastating eye infection and potentially blinding medical emergency.^[^ ^1^, ^2^ ^]^ The incidence of endophthalmitis following cataract surgery is approximately 0.1%.^[^ ^3^, ^4^ ^]^ The BE, as a rare complication, could lead to irreversible blindness and even eyeball losing if lack of timely and effective treatment.^[^ ^5^ ^]^ The visual function of BE patient is determined by the timely treatment, toxicity of invaded bacteria, and the severity of intraocular inflammation activated by bacteria and other components.^[^ ^5^, ^6^ ^]^ At present, BE is commonly treated with empirical intravitreal injection of vancomycin in combination with other antibiotics, and vitrectomy is necessary in some severe cases.^[^ ^4^ ^]^ Even though the antibiotics could be easily conducted by an intravitreous injection, overuse or misuse of the antibiotics would result in drug resistance of pathogens, even with the emergence of super bacteria.^[^ ^7^ ^]^ Besides, the vancomycin‐associated hemorrhagic occlusive retinal vasculitis has been reported in some BE cases after intravitreous vancomycin injection, leading to extremely poor visual acuity.^[^ ^8^ ^]^ Due to the potential drug resistance and limited effect on some bacteria of antibiotics, long hospitalization period and repeated injections are needed, resulting in time‐consuming and money‐wasting for some patients. Therefore, it is of utmost urgency to develop alternative antibacterial therapeutics of BE to solve the aforementioned problems.

Photodynamic therapy (PDT) has shown a great potential in the treatment of drug‐resistant bacterial infections.^[^ ^9^ ^]^ Photosensitizers (PSs) are activated to produce reactive oxygen species (ROS) to kill pathogens under light irradiation during PDT.^[^ ^10^, ^11^, ^12^ ^]^ The PDT strategy reveals low cytotoxicity, negligible antimicrobial resistance, and spatiotemporal accuracy over conventional therapy, showing outstanding advantages in antibacteria treatment.^[^ ^13^, ^14^ ^]^ Importantly, PDT is very suitable for ocular infectious therapy due to the excellent light transmission property of eyeball compared with other tissues.^[^ ^15^ ^]^ PDT has been showed great antibacterial ability and biocompatibility for BE treatment.^[^ ^16^ ^]^ However, most of traditional PSs have the problem of aggregation‐caused quenching in the aggregate state due to *π*–*π* stacking, resulting in unaccepted weak emission and poor ROS production, which hinders their clinic applications.^[^ ^17^, ^18^ ^]^

In recent few years, aggregation‐induced emission luminogens (AIEgens) have successfully showed the great potential as promising PSs.^[^ ^19^, ^20^, ^21^, ^22^, ^23^, ^24^ ^]^ Because of the restriction of intramolecular motions, AIEgens could be induced to give intense emission and generate efficient ROS in the aggregates, while show weak emission in the monomer state.^[^ ^25^ ^]^ Some photodynamic materials have been used in bacteria killing strategy of wounds, such as semiconductors and inorganic nanoparticles.^[^ ^26^, ^27^, ^28^ ^]^ However, AIEgens with proper design can possess moderate water solubility, high ROS generation efficiency, low dark toxicity but prominent phototoxicity, and targets specificity.^[^ ^29^ ^]^ Due to the photobleaching resistant ability and high emission efficacy, AIEgens have been applied in biomedical imaging of cells, bacteria, tissues, and animals.^[^ ^30^ ^]^ It has been reported that AIEgens can eliminate drug‐resistant bacteria infection and ablate cancer cells by regulating incubation time, leaving normal cells unaffected.^[^ ^29^ ^]^ Besides, other studies showed that cationic AIEgens could specifically discriminate Gram‐positive bacteria over mammalian cells due to membrane potential difference.^[^ ^21^, ^31^, ^32^ ^]^ Recently, Peng and Zhou et al. reported that positive charged AIEgens could treat keratitis, which exhibited excellent photodynamic antibacterial ability and biosafety of ocular applications.^[^ ^33^, ^34^ ^]^ According to the previous studies, proper design of cationic AIEgens could specifically discriminate and kill bacteria by PDT over normal cells.

**Scheme 1:**
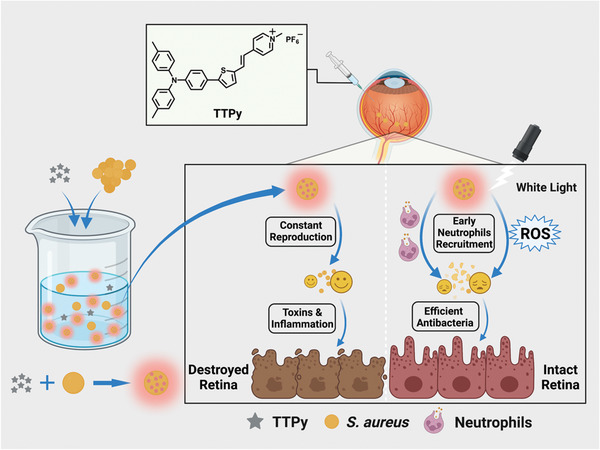
Schematic illustration of TTPy for rapid discrimination and excellent photodynamic antibacteria. Under white light irradiation, efficient ROS generation and early immune response induced by TTPy protected the retina from toxins and inflammation‐related damage.

In this contribution, a kind of cationic AIEgens with suitable hydrophobicity, named triphenylamine thiophen pyridinium (TTPy), has been synthesized and utilized for antibacterial study and BE treatment (**Scheme** **1**). *Staphylococcus aureus* (*S. aureus*), which usually caused severe acute inflammation and poor visual outcome, was used to explore the bacteria killing ability of TTPy.^[^ ^5^ ^]^ As expected, driven by electrostatic and hydrophobic interaction, TTPy could rapidly discriminate and bind to *S. aureus*. Upon addition of light irradiation, a lot of ROS was generated to kill *S. aureus* efficiently without obvious cytotoxicity at a relative low concentration. Interestingly, the high killing efficacy of TTPy caused an early innate immune response, a rapid recruitment of neutrophils, and diminishment of subsequently inflammatory burst in rat models of BE. This not only inhibited the spread of infection, but also protected retina from inflammation‐related bystander tissue damage. In addition, compared with commercially used Rose Bengal (RB), TTPy with high bacteria killing efficacy also showed good biocompatibility of retina and effectively avoided vision loss, which provided a new strategy for BE treatment.

## 2 Results and Discussion

### 2.1 Photophysical Property

As shown in the inserted picture of Figure S1 (Supporting Information), TTPy was synthesized and purified according to the previous report.^[^ ^35^ ^]^ TTPy was diluted to different concentrations to analyze the photophysical property. As shown in Figure S1A (Supporting Information), the red fluorescence was observed with the naked eye when *S. aureus* was incubated with TTPy for 15 min under 365 nm UV light, suggesting its good affinity and bioimaging ability to *S. aureus*. Subsequently, pure TTPy photoexcited by 489 nm showed the emission maximum wavelength at around 610 nm, without obvious difference of PL intensity within various concentrations (Figure S1B, Supporting Information). Upon incubation with *S. aureus* for 15 min, the PL intensities are conspicuously enhanced with a concentration dependent redshift of the emission maximum. This might be caused by the different aggregate state of TTPy incubated with *S. aureus*. The visible fluorescence images and PL spectra indicated that TTPy possessed strong affinity to *S. aureus*, showing its promising capability as an agent for bacterial imaging.

### 2.2 Photodynamic Antibacterial Study

**Figure 1:**
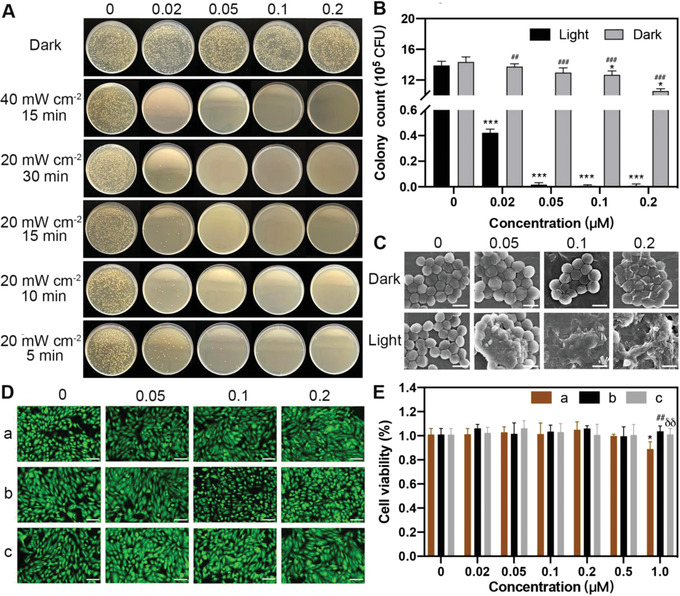
A) Representative photographs of *S. aureus* treated by TTPy at a concentration range of 0–0.2 × 10^−6^ m in different irradiation conditions. B) The surviving colony count of *S. aureus* treated by TTPy in light condition (20 mW cm^−2^) for 15 min (* for comparison among different concentrations of TTPy [0.02, 0.05, 0.1, 0.2 × 10^−6^ m) versus TTPy (0 × 10^−6^ m) in light and dark conditions, # for comparison between light and dark at the same concentration). C) SEM images of *S. aureus* treated by different concentrations of TTPy with light (20 mW cm^−2^) and without light irradiation for 15 min (scale bar: 1 µm). D) Live/dead staining images of ARPE‐19 cells, (a) with light after incubation with TTPy for 15 min and (b) without light after incubation with TTPy for 15 min (c) treated by TTPy for 24 h without light (scale bar: 100 µm). E) Cell viability of ARPE‐19 cells treated by TTPy at 0–1.0 × 10^−6^ m in different irradiation conditions, a, b, and c were as previously described (* for comparison among different concentrations of TTPy (0.02, 0.05, 0.1, 0.2, 0.5, or 1.0 × 10^−6^ m) versus TTPy (0 × 10^−6^ m) in group a, b, and c, # for comparison between group a and b, and *δ* for comparison between group a and c under the same concentration).

Encouraged by the excellent affinity of TTPy to *S. aureus*, traditional spread plate method was used to assess the photodynamic killing effect. Firstly, as shown in **Figure** **1**A, there was nearly no surviving bacterial colony with a relative low concentration of 0.02 × 10^−6^ m in an irradiation condition of 40 mW cm^−2^ for 15 min according to the previous study.^[^ ^21^ ^]^ Afterward, the excellent killing effect was also observed in light condition of a lower power (20 mW cm^−2^) and a longer irradiation time (30 min). To confirm the optimal light condition, less irradiation time (5, 10, and 15 min) were subsequently conducted. Amazingly, *S. aureus* was killed effectively with nearly no surviving colony in the presence of 0.05 × 10^−6^ m TTPy and light irradiation (20 mW cm^−2^) for 10 and 15 min (Figure S2, Supporting Information, and Figure 1B). To obtain the best killing effect, the illumination condition of 20 mW cm^−2^ for 15 min was used for the following study. Additionally, the antibacterial efficiency of higher concentrations of TTPy was also evaluated. As is shown in Figure S3 (Supporting Information), the intrinsic toxicity was significantly observed at 1.0 × 10^−6^ m of TTPy. In addition, scanning electron microscopy (SEM) was further employed to observe the bacterial morphology changes. As shown in Figure 1C, *S. aureus* incubated with pure TTPy or light remained intact with well‐defined and clear cell wall borders. Upon addition of light and TTPy, *S. aureus* were tremendously destroyed without irregular morphology, which directly demonstrated the high efficiency in killing *S. aureus*. From the foregoing, TTPy exerted antibacterial effect by damaging the regular shape of bacteria. Therefore, how does TTPy damage *S. aureus*?

### 2.3 Antibacterial Mechanisms of AIEgens

Afterward, the mechanisms of TTPy killing bacteria were explored by detecting the ROS generation of TTPy and zeta potentials of *S. aureus*. As shown in Figure S4 (Supporting Information), the ROS generation of TTPy at 0.1 × 10^−6^ m was significantly increased under white light irradiation (20 mW cm^−2^) within 15 min. It has been demonstrated that AIEgens can generate efficient ROS in aggregation state, which is possibly caused by the promoted energy transfer from the lowest excited singlet state (S1) to the lowest triplet state (T1) due to the prohibition of energy dissipation through nonradiative channels and aggregation‐induced intersystem crossing (AI‐ISC).^[^ ^36^, ^37^ ^]^ TTPy, a kind of cationic AIEgens, was believed to benefit from the small singlet–triplet energy gaps (0.565 eV), facilitating the induced ISC process from S1 to T1 and significantly improving the yield of the triplet excited state.^[^ ^35^ ^]^ Moreover, the previous study showed TTPy had an excellent performance in generating singlet oxygen, which played an important role in antibacterial process.^[^ ^21^ ^]^ In addition, by comparison, Figure S4 (Supporting Information) showed that RB has a comparative ROS generation with TTPy at the same condition in vitro.

To verify the affinity of TTPy to *S. aureus*, zeta potentials of *S. aureus* incubated with different concentrations of TTPy were measured. Generally, the cell walls of Gram‐positive bacteria are consisted with interconnected peptidoglycan layers, which are intercalated by negatively charged teichoic acids.^[^ ^38^ ^]^ Here, the zeta potential value of pure *S. aureus* was −11.20 ± 0.15 mV, while the values were increased to −9.12 ± 0.10, −7.54 ± 0.42, and −5.42 ± 0.96 mV after incubated with TTPy (0.02, 0.05, and 0.1 × 10^−6^ m) (Figure S5, Supporting Information). Therefore, the cationic charged TTPy was driven to the negatively charged teichoic acids on the surface of *S. aureus* owing to electrostatic interaction. Furthermore, TTPy could pass through the lipid membrane and entered the *S. aureus* due to the good hydrophobicity. Upon the addition of light, the generated abundant ROS might damage the cell wall, resulting in the irregular shape and death of *S. aureus*. In addition, since surfaces of normal cells are also negatively charged, co‐culture of *S. aureus* and human retinal pigment epithelial (ARPE‐19) cells was also performed to further confirm the specific target of TTPy to bacteria. As shown in Figure S6 (Supporting Information), the bright fluorescence was significantly observed in *S. aureus*, while nearly negligible fluorescence signals in ARPE‐19 cells, due to their surface charge discrepancy. Collectively, these results fully confirmed the highly selective specificity and killing efficiency of TTPy to *S. aureus*, implying its promising potential as antibacterial substance in vitro.

### 2.4 Biocompatibility Evaluation

Prior to biological applications, biocompatibility evaluation is considerably crucial, especially for ocular injection in the present study. Since AIEgens were injected into the vitreous cavity of eyes, common ARPE‐19 cells in the retina were used to estimate the biological security, which were vital to visual function. As shown in Figure 1D, the live/dead staining assay of ARPE‐19 cells did not show significant change in green and red fluorescence after incubation with TTPy with and without light (20 mW cm^−2^, 15 min) at a concentration range of 0–0.2 × 10^−6^ m, indicating the integrity of cell membranes and low cytotoxicity of TTPy. Moreover, CCK‐8 was used to further detect cell viability, which suggested that the cell viabilities of ARPE‐19 cells had no significant decrease at concentrations below 0.5 × 10^−6^ m both in dark and light conditions (20 mW cm^−2^, 15 min), and even after incubation with TTPy without light for 24 h (Figure 1E). However, an obvious decrease of cell viability was observed at 1.0 × 10^−6^ m in light irradiation. Surprisingly, no cytotoxicity was observed at the concentration range of 0–1.0 × 10^−6^ m in dark condition, implying the potential of TTPy as a promising PS for PDT.

In addition, TTPy did not obviously change the cell morphology and density of ARPE‐19 cells (Figure S7, Supporting Information). Subsequently, western blot and flow cytometry analysis were used to evaluate the apoptotic function of cells. Surprisingly, the expressions of Bcl‐2 and Bax, two apoptosis‐associated proteins, were not apparently affected by treatment with TTPy (Figure S8A–C, Supporting Information). Besides, TTPy did not also result in increasing apoptosis cells in different irradiation conditions (Figure S8D,E, Supporting Information).

Moreover, human corneal epithelial (HCE‐T) cells were used to further evaluate the biosafety of TTPy. As shown in Figure S9 (Supporting Information), HCE‐T cells presented good cell viability below 0.1 × 10^−6^ m in dark and light conditions. The hemolysis ratios were all less than 1% at different concentrations of TTPy (Figure S10, Supporting Information). In summary, these findings demonstrated that TTPy possessed good biocompatibility and hemocompatibility, which was suitable for the following study.

### 2.5 Treatment of Bacterial Endophthalmitis

The BE model was established by injecting 100 colony forming unit (CFU) of *S. aureus* into the vitreous cavity of SD rats eyes. Due to the trauma of intraocular injection, the treatment was performed only once, just after 1 h post injection of *S. aureus* in the overall process of 7 d. Herein, the commercially available RB was used as a contrast to better evaluate the therapeutic effect of TTPy in vivo. For the experimental groups, the infected eyes were injected 10 µL phosphate buffer saline (PBS) (group Control), RB with light (group RB+Light), TTPy without light (group TTPy+Dark), and TTPy with light (group TTPy+Light). The uninfected rats were set as normal group. To evaluate the antibacterial efficiency and inflammation response, several indexes were involved and examined, including ocular manifestation, histopathology of whole eyeball, vitreous bacterial colonies, ultrasound examination, and expression of inflammation‐related cytokines.

**Figure 2:**
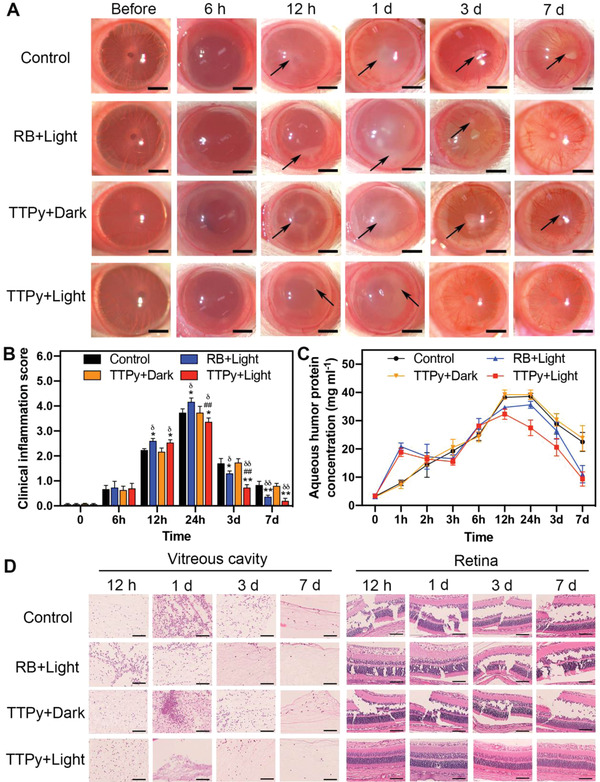
A) Representative photographs of rat eyes under slit lamp before and at 6 h, 12 h, 1 d, 3 d, and 7 d after treatment (scale bar: 1 mm). B) The clinical inflammation score of rat eyes and C) the concentration of aqueous humor protein after different treatments. H&E images of D) vitreous cavity and E) retina at 12 h, 1 d, 3 d, and 7 d after different treatments (scale bar: 100 µm, * for comparison among group RB+Light, TTPy+Dark, TTPy+Light vs group Control at the same time point, # for group RB+Light vs group TTPy+Light, *δ* for group RB+Light, TTPy+Light vs group TTPy+Dark).

As shown in **Figure** **2**A, no obvious inflammation was observed under slit‐lamp at 6 h after ocular treatment due to the immune privilege of vitreous cavity which allowed bacterial replication and limited inflammation.^[^ ^5^ ^]^ At 12 h, white fibrinous exudation (black arrow) increased at the edge of the pupil, with dilated iris vessels and caligo pupillae. The eyes treated with TTPy and RB in light condition showed more exudate and higher clinical inflammation score (CIS) than other groups (Figure 2B). The results suggested that the inflammation was more severe after photodynamic treatment of TTPy and RB at 12 h, which might indicate that the antibacterial behavior of TTPy could lead to an earlier inflammation response. With the continuation of infection and inflammation, the CIS of four groups peaked at 1 d post‐treatment, along with obvious ocular manifestation of corneal edema, fibrinous exudations in whole anterior chamber, obscure iris vessels and caligo pupillae. Surprisingly, a relatively clearer cornea, less exudation and lower CIS than other groups were observed in group TTPy+Light, indicating that the photodynamic treatment of TTPy could well suppress the infectious progress and alleviate clinical inflammation in the meantime. In contrary, the obvious hypopyon, a sign of severe inflammation, appeared in eyes treated with RB at 1 d post‐treatment, which might be ascribed to the non‐specificity of RB to bacteria and normal cells, leading to dual tissue damage and further vigorous inflammation in the eye. Subsequently, inflammation of all eyes gradually relieved at 3 d post‐treatment due to the clearance of immune system. However, the fibrinous exudation in anterior chamber had been totally absorbed in eyes of group TTPy+Light, while the effusion was still observed in the pupil area of other groups, suggesting that the infection and inflammation were well‐controlled in the attendance of TTPy and irradiation. At 7 d, the anterior chamber recovered to clear with only dilated iris vessels after photodynamic treatment of TTPy and RB. However, a small amount of fibrin remained unabsorbed in group Control and TTPy+Dark. Collectively, these results preliminarily suggested that TTPy had prominent performance in antibacterial efficacy and inflammation alleviation in rat models of BE.

Generally, the eye is protected from inflammatory cells by blood ocular barrier (BOB), including blood aqueous barrier (BAB) and blood retinal barrier (BRB), which can be damaged by inflammation during endophthalmitis, resulting in fibrin, albumin, and neutrophils infiltrate to aqueous and vitreous humor from blood.^[^ ^39^ ^]^ To evaluate the changes of BOB in BE, the concentration of aqueous humor protein (AHP) was measured. As Figure 2C showed, the AHP concentrations treated by TTPy and RB with light were significantly higher than that of other groups at 1 h post‐treatment, and subsequently decreased at 2–3 h. The temporary increase of AHP might be ascribed to the ROS generation after irradiation, resulting in transitory inflammation and elevation of the vascular permeability of BOB.^[^ ^40^ ^]^ This indirectly suggested the high efficiency of ROS generation of AIEgens after irradiation in vivo. After 12 h, AHP concentrations of group Control and TTPy+Dark were significantly higher than that of group TTPy+Light, which might be due to the toxins secreted by proliferating *S. aureus* leading to inflammation and breakdown of BOB. However, the obvious difference was also observed between eyes treated by TTPy and RB with light after 12 h, which might due to the non‐selectivity of RB to bacteria and retinal cells, aggravating the inflammatory response and leading to partial albumin infiltrated through the damaged BOB. At 7 d, the AHP concentration recovered to the early baseline level in eyes treated by TTPy and light, suggesting that the breakdown of BOB was milder than other eyes. In summary, these results showed that TTPy had efficient antibacterial capacity and made a slight breach to intraocular barriers, which was important to maintain the balance of intraocular environment.

### 2.6 Histological Analysis

At 12 h, 1 d, 3 d, and 7 d post‐treatment, the rats were euthanized and the eyeballs were removed for hematoxylin and eosin (H&E) staining to observe the ocular architecture. A large number of inflammatory cells and fibrins infiltrated to the vitreous cavity (Figure 2D, left), anterior chamber (Figure S11, Supporting Information), and ciliary body (Figure S12, Supporting Information) of all eyes after infection, compared with normal eyes (Figure S13, Supporting Information). However, vitreous cavity of eyes treated by TTPy and RB with light showed more infiltrating inflammatory cells than other groups at 12 h (Figure 2D, left, and Figure S14, Supporting Information). It is known that bacterial components, such as peptidoglycan and teichoic acids, which constitute 70% of the weight of cell wall, are recognized by pattern recognition receptors expressed on immune cells and epithelial cells to initiate innate immunity against pathogens in BE.^[^ ^41^, ^42^ ^]^ Here, more peptidoglycans were exposed to ocular immune cells after *S. aureus* killed by ROS, which might lead to more inflammatory cells recruited to the infected sites at an early stage, assisting to clear the surviving *S. aureus*. Contrarily, there was more obvious influx of inflammatory cells in group Control and TTPy+Dark at 1 d post‐treatment, which might be due to a higher concentration of toxins secreted by numbers of replicated *S. aureus* leading to a robust inflammation in the eye. Toxins combined severe inflammation resulted in irreversible retinal damage, including entire retinal detachment and dissolution of retinal cells (Figure 2D, right). Amazingly, few neutrophil infiltration and fibrinous exudation were observed in eyes treated by TTPy and light at 1, 3, and 7 d post‐treatment, which protected the delicate retina from bystander tissue damage. Moreover, although RB could kill bacteria under light, it still caused severe retinal damage. The nonselectivity of RB to bacteria and normal cells was further verified by frozen section staining of eyeballs after intravitreal injected with TTPy and RB (Figure S15, Supporting Information). In summary, these results demonstrated that the antibacterial ability of AIEgens could lead to an early inflammation response and subsequently decrease the peak of robust inflammation, which protected the retina from invasion of bacteria and inflammation‐related bystander damage.

### 2.7 Ultrasound Examination

**Figure 3:**
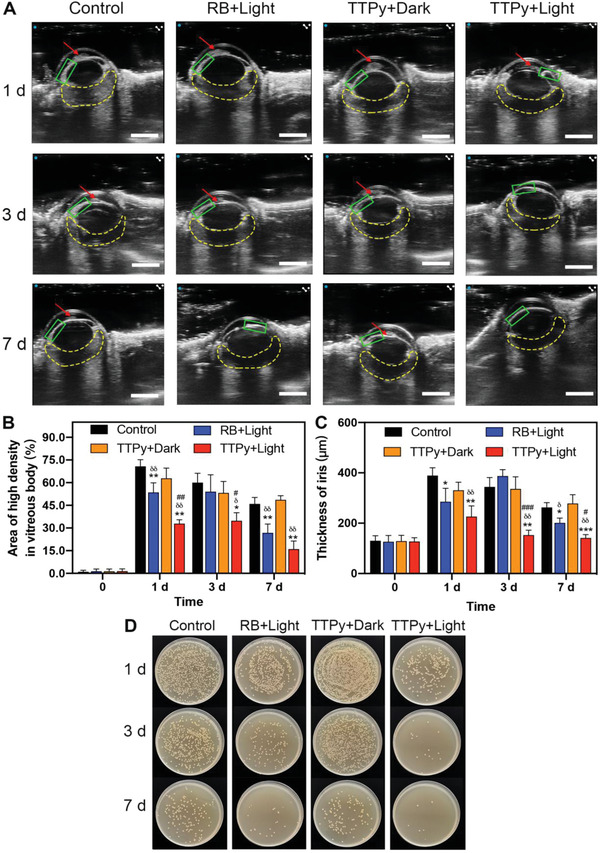
A) Representative ultrasound images of rat eyes at 1, 3, and 7 d after different treatments, red arrows: fibrinous exudate in anterior chamber, green rectangle: iris, yellow dotted lines: vitreous cavity (scale bar: 500 µm). B) Area of high density in vitreous cavity C) thickness of iris and D) bacterial cultures on agar plates from the corresponding vitreous humor at different time points (* for group RB+Light, TTPy+Dark, TTPy+Light vs group Control at the same time point, # for group RB+Light vs group TTPy+Light, *δ* for group RB+Light, TTPy+Light vs group TTPy+Dark).

To obtain a comprehensive evaluation of inflammatory changes, B‐ultrasound, a routine examination for clinical diagnosis and treatment of endophthalmitis, was used to observe the whole eyeballs in vivo.^[^ ^43^, ^44^ ^]^ As shown in **Figure** **3**A, the high density of fibrinous exudate in the anterior chamber (red arrow), vitreous cavity (yellow dotted line), and the thickening iris (green rectangle) were observed in all eyes at 1 d post‐treatment. However, eyes treated by TTPy and light had significant less area of high density and thinner iris thickness than other groups within 7 d (Figure 3B,C), which indicated that TTPy could well control the infection and relieve the inflammation. In addition, the surviving bacteria were significantly reduced after treated by TTPy and RB with light compared with other groups (Figure 3D and Figure S16, Supporting Information). Even so, the obvious difference was also observed between the two groups with photodynamic treatment, which showed TTPy performed better in killing bacteria and controlling inflammation than RB in vivo, due to the nonspecificity of RB to bacteria and cells. Besides, there was no significant difference of *S. aureus* counts between group Control and group TTPy+Dark at 1, 3, and 7 d (all *P* > 0.05) after treatment. Collectively, these findings were consistent with the previous results, showing TTPy a better promising potential as an antibacterial agent than commercially used RB.

### 2.8 Immune Response

It is established that the innate immune system is the first line of host defense against invading pathogens.^[^ ^5^ ^]^ The robust inflammation mediated by inflammatory cells and mediators is a hallmark of endophthalmitis. The proinflammatory cytokines are vital signaling mediators in detecting invading bacteria, recruiting immune cells, mainly neutrophils in acute intraocular infection.^[^ ^3^ ^]^ To further evaluate the innate immune response after treatment, the vitreous inflammatory cytokines, CXCL1, TNF‐*α*, IL‐1*β*, and IFN‐*γ*, were detected by enzyme‐linked immunosorbent assays (ELISA). CXCL1, TNF‐*α*, and IL‐1*β* are typical cytokines in BE infected by *S. aureus* which contribute to the breakdown of BRB and the early recruitment of neutrophils.^[^ ^45^ ^]^

**Figure 4:**
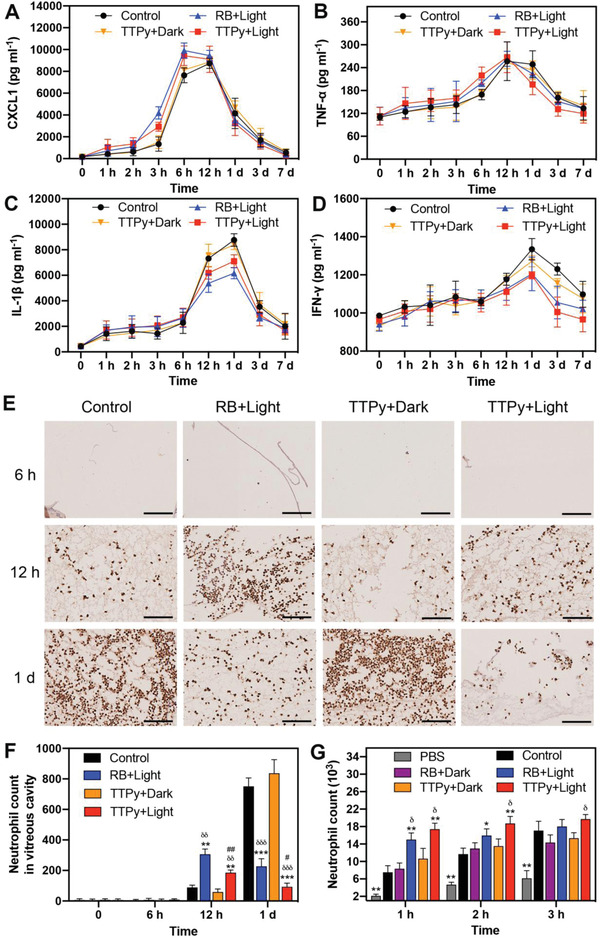
Levels of different cytokines in corresponding vitreous cavity after different treatments A) CXCL1, B) TNF‐*α*, C) IL‐1*β*, and D) IFN‐*γ*. E) Immunohistochemical staining analysis of MPO at 6 h, 12 h, and 1 d after different treatments (scale bar: 100 µm). Neutrophils count F) in corresponding vitreous cavity and G) in transwell assay (* for group RB+Light, TTPy+Dark, TTPy+Light, and PBS versus group Control at the same time point, # for group RB+Light vs group TTPy+Light, *δ* for group RB+Light, TTPy+Light vs group TTPy+Dark).

As shown in **Figure** **4**A–D, the levels of all inflammatory factors increased to a peak and then recovered to the baseline after infection as the previous study reported.^[^ ^46^ ^]^ CXCL1, the earliest one reaching the peak at 6–12 h, was significantly higher in eyes photodynamic treated by TTPy (*P* = 0.023) and RB (*P* = 0.004) at 3 and 6 h (*P* = 0.027, *P* = 0.012, respectively) than group Control (Figure 4A), which could recruit more neutrophils to the site of infection. This might be due to more peptidoglycans in cell walls were exposed to innate immune system after *S. aureus* killed by ROS, resulting in a higher concentration of cytokines released to vitreous cavity at 12 h. However, the injury of retinal cells caused by the non‐selectivity of RB might lead to higher CXCL1 level at 3 h compared with TTPy. TNF‐*α* is a potent chemokine of inflammatory cells in ocular infection, expressed by multiple cell types, including macrophages, neutrophils, RPE, and Müller cells in the eye.^[^ ^47^ ^]^ The increase of TNF‐*α* could facilitate vascular permeability and cytokines IL‐1 production, resulting the breakdown of BRB.^[^ ^48^ ^]^ Consistent with CXCL1, higher level of TNF‐*α* in eyes treated by TTPy with light was also observed at 6 h compared with group Control (*P* = 0.028), which could result in increased neutrophils and mononuclear cells recruitment in the vitreous chamber (Figure 4B). Surprisingly, the concentrations of CXCL1 and TNF‐*α* decreased more rapidly to the baseline in group TTPy+Light, which suggested less infiltrated neutrophils due to the rehabilitation of BRB. Considering that RB is more likely to attach the retinal cells, the photodynamic treatment with RB might cause more retinal damage and the breakdown of BRB, resulting in higher level of TNF‐*α* and more infiltrated neutrophils than TTPy after 1 d post‐treatment.

It has been demonstrated that *α*‐toxins secreted by *S. aureus* could induce inflammatory response via IL‐1*β* and lead to bystander tissue damage.^[^ ^47^ ^]^ Here, IL‐1*β* was found significantly higher in eyes of group Control and TTPy+Dark at 12 h and 1d than other groups (all *P* < 0.05) (Figure 4C), and this might be caused by large amounts of *α*‐toxins secreted by proliferating *S. aureus* in immune privileged vitreous chamber after injection. In addition, IFN‐*γ* is correlated with the recruitment of macrophages and lymphocytes in late course of BE.^[^ ^5^ ^]^ It was reported that neutrophils were decreased with the decline of CXCL1, TNF‐*α*, and IL‐1*β* levels after their respective peaks, while the infiltration of macrophages and lymphocytes were increased mediated by IFN‐*γ*.^[^ ^46^ ^]^ In our study, lower IFN‐*γ* level in eyes treated by TTPy and light after 1 d post‐treatment than group Control and TTPy+Dark demonstrated less infiltration of macrophages and lymphocytes to the vitreous cavity (all *P* < 0.05) (Figure 4D). This might be ascribed to efficient antibacteria of AIEgens and phagocytosis of early recruited neutrophils, which cleared most surviving *S. aureus* and successfully suppressed the infection in the early stage.

As is well known, neutrophils are the first inflammatory cells recruited to the eyeball in BE.^[^ ^3^, ^49^ ^]^ Therefore, the immunochemical staining of myeloperoxidase (MPO) was performed to observe the infiltration of neutrophils in an early stage of inflammation. As shown in Figure 4E, neutrophils were not found in vitreous cavity before 6 h post‐treatment. This could be due to the immune privilege of vitreous cavity, which is permissive for bacterial replication, toxins production and limits inflammation in the eye.^[^ ^5^ ^]^ The number of neutrophils in eyes of photodynamic treated by TTPy and RB were much more than other groups at 12 h (Figure 4F), which was consistent with previous results, and verified again that the antibacterial behavior of TTPy caused a faster immune response. However, an unimpeded influx of neutrophils and mediators into the vitreous cavity caused robust inflammation and terrible bystander tissue damage at 1 d in group Control and TTPy+Dark. In addition, due to the difference of microenvironment between in vivo and in vitro, we conducted a transwell assay to evaluate the migration of neutrophils when encountered with inflammatory signals in vitro. As shown in Figure 4G, more neutrophils were obviously migrated to the bottom well of *S. aureus* incubated with TTPy and RB under light at 1 h. After an incubation for 3 h, there was no significant difference of the migrated neutrophils number among those groups. Collectively, these findings demonstrated that the antibacterial behavior of TTPy could result in an early neutrophils recruitment to the infectious site of vitreous cavity, which not only limited the spread of infection but also protected the delicate retina from inflammation‐related bystander damage.

### 2.9 In Vivo Biosafety

As shown in Figure S17 (Supporting Information), the animal experiments did not cause obvious damage to visceral organs including heart, liver, spleen, lung, and kidney, and also the hepatic and renal function (Figure S18, Supporting Information). Besides, the AHP concentration had no significant fluctuation after intravitreal injection of pure TTPy (Figure S19, Supporting Information). After treatment, the weight of rats within 7 d changed within an acceptable range (Figure S20, Supporting Information). These results showed TTPy a great biocompatibility and promising antibacterial potential in BE.

## 3 Conclusion

In conclusion, a bacteria‐specific PDT strategy based on TTPy was utilized for antibacterial study on BE. TTPy could selectively combine to bacteria over normal ocular cells due to its cationic charge and suitable hydrophobicity. The extraordinary ROS generation property of TTPy under light irradiation was induced by its small singlet–triplet energy gap. Meanwhile, TTPy displayed prominent killing efficiency and great cytocompatibility, which indicated its great potential in vivo photodynamic antibacterial research. As expected, TTPy presented excellent antibacterial effect in rat BE infected by *S. aureus*. Interestingly, the efficient antibacterial behavior of TTPy triggered an early immune response to the intraocular infection. The early influx of neutrophils limited the replication of surviving *S. aureus* and reduced toxins production, which not only suppressed the infection and alleviated the robust inflammation, but also protected the delicate retinal cells from inflammation‐related bystander damage. Importantly, TTPy showed better bacteria killing performance than commercially used RB, protected the retinal architecture, and greatly saved the visual function in the meantime. Therefore, the photodynamic antibacterial strategy based on AIEgens may provide a guidance for further preclinical research on infection and present great potential in clinical applications.

## 4 Experimental Section

### In Vitro Antibacterial Test

TTPy was synthesized and purified according to the previous report.^[^ ^35^ ^]^ Traditional spread plate method was used to assess antibacterial activity in vitro. *S. aureus* suspension was incubated with increasing concentrations of TTPy (0, 0.02, 0.05, 0.1, 0.2, 0.5, 1.0, and 2.0 × 10^−6^ m) for 15 min and then placed in dark and light conditions for another certain time (5, 10, 15, 30 min), respectively. The mixture was diluted at a ratio of 1:500, and 100 µL of dilution was spread evenly onto an agar plate for 24 h culture at 37 °C. The number of surviving bacterial colonies was counted.

In addition, SEM was used to intuitively observe the morphologies of *S. aureus* after different treatments. *S. aureus* suspension was incubated with different TTPy concentrations (0, 0.02, 0.05, and 0.1 × 10^−6^ m) for 15 min and then exposed in dark or light conditions for another 15 min, respectively. Then the bacteria were washed with PBS for 3 times followed by fixed with 4% paraformaldehyde overnight. After centrifuged for 10 min at 7100 rpm, the supernatant was removed, and the bacteria sediment were dehydrated with 40%, 70%, 90%, and 100% ethanol in a gradient manner for 6 min, respectively. The bacteria suspension resuspended with 100% ethanol was dripped on the aluminum foil and dried at room temperature. The images were taken on a scanning electron microscope.

### ROS Measurement

The ROS generation was measured with Cellular Reactive Oxygen Species Detection Assay Kit. DCFH‐DA solution (1 µL, 10 × 10^−3^ m) was added to TTPy solution (1 mL, 0.1 × 10^−6^ m). The obtained mixture solution was added to a 96‐well black plate, then irradiated with a white light lamp (20 mW cm^−2^) at intervals of 1 min until 15 min. After each irradiation interval, the absorbance of solution was recorded immediately on a Thermo Scientific Microplate Reader (*λ*_ex_ = 480 nm, *λ*_em_ = 525 nm). The absorbance change of DCFH‐DA, PBS alone with 15 min irradiation time was also measured as control.

### Zeta Potential Measurements

*S. aureus* was firstly collected (4000 rpm, 5 min) to remove medium, and resuspended with PBS. Then the *S. aureus* suspension was incubated with different concentrations of TTPy (0, 0.02, 0.05, and 0.1 × 10^−6^ m) for 15 min. Then a Malvern Zetasizer was used to measure the Zeta potential of *S. aureus* suspension.

### Co‐Culture of Bacteria and Cells

*S. aureus* and cells were co‐cultured with TTPy to evaluate the selective affinity to bacteria over cells. ARPE‐19 cells were cultured in confocal dishes at 1 × 10^5^ cells per dish overnight for adhesion in a 5% CO_2_ incubator at 37 °C. *S. aureus* suspensions and TTPy with different concentrations (0, 0.02, 0.05, and 0.1 × 10^−6^ m) were added into the dish in order. After incubation for 15 min, images were taken under Zeiss laser scanning confocal microscope (*λ*_ex_ = 488 nm).

### Cytotoxicity

ARPE‐19 cells and HCE‐T cells were used to evaluate the cytotoxicity of TTPy. Cells were cultured in DMEM/F12 (1:1) mixed medium supplemented with 10% fetal bovine serum and 1% penicillin and streptomycin. Besides, 10 ng mL^−1^ human epidermal growth factor and 5 µg mL^−1^ insulin human recombinants were, respectively, added to the culture medium of HCE‐T cells. The cells without any treatment were grown in a 5% CO_2_ incubator at 37 °C as control.

ARPE‐19 cells and HCE‐T cells were separately seeded in 96‐well plates at 1 × 10^4^ cells per well and incubated overnight for adhesion. TTPy in increasing concentrations (0–1.0 × 10^−6^ m) were added to each well for 15 min incubation and then treated in light (20 mW cm^−2^, group a) and dark conditions (group b) for another 15 min at room temperature, respectively. Then cells were incubated for another 24 h after the medium was replaced with 100 µL fresh medium. Considering the relatively confined chamber of eyeball, the cells were incubated with different concentrations of TTPy for 24 h under dark (group c) to evaluate its intrinsic cytotoxicity. Next, cell viability was analyzed by a microplate reader at 450 nm after an incubation with 10% CCK‐8 reagent for 2 h. The absorbance was proportional to the number of living, metabolically active cells.

The live/dead staining of ARPE‐19 cells was used for further evaluation of cytotoxicity of TTPy. After incubated overnight in six‐well plates, ARPE‐19 cells were incubated with different concentrations of TTPy under different irradiation conditions as described above, and then stained with Calcein/PI kit according to the given protocol. Finally, the images were taken on a Nikon fluorescence microscopy.

### Animal Experiments of Antibacterial Test

The animal experiments protocol in this paper were approved by Animal Research Ethical Committee of Shanghai Tenth People's Hospital affiliated to Tongji University (SHDSYY‐2021‐1902, Shanghai, China). SD rats (7–8 weeks, 180–200 g, male) were chosen to establish the animal models of BE and maintained under pathogen‐free conditions. All rats were anesthetized by intraperitoneal injection with 3.5% pentobarbital sodium of 0.4 mL. Prior to any injections, eyes were examined to confirm the absence of any abnormalities with a slit lamp microscope. Before intravitreous injection of bacterial solution, 10 µL of the aqueous humor was taken out to avoid high intraocular pressure and solution leakage. Sterile tropicamide eye drops and 0.5% Alcaine were used for pupillary dilation and local anesthesia of the ocular surface. 10 µL *S. aureus* suspension (100 CFU) was injected into the right eye with a microsyringe connected to a 30G needle.^[^ ^49^, ^50^ ^]^ The needle was inserted vertically from the limbus and positioned behind the lens in the vitreous capacity. After pushing, the needle should be paused a while before pulling. After 24 h, the formation of BE was observed under slit lamp.

All rats were intravitreous injected with *S. aureus* solution and divided randomly into four groups for the following study. After injected with *S. aureus* suspension for 1 h, the infected rats were injected with 10 µL 1× PBS (group Control), 0.1 × 10^−6^ m RB with light (group RB+Light), 0.1 × 10^−6^ m TTPy without light (group TTPy+Dark) or with light (group TTPy+Light), respectively. After an incubation for 15 min, eyes were treated in dark or light conditions (20 mW cm^−2^ power) for 30 min. In addition, the uninfected rats were set as normal group without any treatment. Photographs of the ocular surface at different time points were captured to observe the infection and inflammation. The clinical inflammation score was used to quantify the severity of inflammation.

The aqueous humor and vitreous humor were obtained at different time points after treatment (1 h, 2 h, 3 h, 6 h, 12 h, 1 d, 3 d, and 7 d). At each time point described, the eyeballs were removed for fast frozen on dry ice. Firstly, the cornea was incised along the corneal limbus and aqueous humor was collected on ice. Then the lens was removed and the vitreous humor was obtained and transferred to conical tubes on ice. Each vitreous sample consisted of the vitreous of three random rats belonging to the same group. The vitreous humor was added to 2.5 µL mg^−1^ PBS before low temperature crushing up. After a centrifuge at 13 000 rpm for 10 min at 4 °C, the supernatant was stored frozen at −80 °C until used. The sediment was resuspended with the same volume of PBS and diluted 1000 times for bacterial culture on agar plate. Rats were humanely killed using a CO_2_ chamber, and both eyes were harvested for histopathology. At 1, 3, and 7 d post‐treatment, ultrasound examination was conducted to observe the inflammation in vivo. Vital organs (heart, liver, spleen, lung, kidney) were obtained before injection and at 7 d post‐treatment.

### Clinical Inflammatory Scores

All rats were graded daily for signs of clinical inflammation with slit‐lamp biomicroscope. The inflammation degree scoring was given according to a previous report.^[^ ^51^ ^]^ Clinical inflammation was scored on a scale of 0 to 4+ in three locations (cornea, iris, anterior chamber).

### Concentration of Anterior Aqueous Protein

The collected aqueous humor was centrifuged at 1800 rpm for 5 min to obtain the supernatant. The total protein concentration was determined by a bicinchoninic acid (BCA) Protein Quantification Kit. The supernatant was diluted 30 times before the measurement.

### Histopathological Analysis

At the various time points, the rats were euthanized and the eyeballs were enucleated to observe the histopathological change. After fixed in fixative solution overnight, the eyes were embedded in paraffin and oriented in the block so that an axis passing through the optic nerve and the center of the cornea was parallel to the surface of the block. All sections were stained with H&E and imaged under an optical microscope.

### Ultrasound Examination

Ocular ultrasonography is routinely performed in endophthalmitis in clinic, which can provide a lot of evidence in diagnosis and inspect the trends of endophthalmitis in vivo.^[^ ^43^, ^44^ ^]^ Especially in patients with media opacity, ultrasound examination is necessary and helpful in the process of endophthalmitis treatment.^[^ ^52^ ^]^ All eyes were examined with B‐ultrasound before and at 1, 3, and 7 d after intravitreous injection for further observation of the vitreous cavity with a small animal ultrasound/photoacoustic imaging system.

### ELISA

To determine the titers of cytokines in the vitreous cavity of rat eyes, ELISA were performed using ELISA kits for CXCL1, TNF‐*α*, IL‐1*β*, and IFN‐*γ* according to manufacturer's instructions.

### Endothelial Transwell Assay

To model the innate immune response, the transwell assay was used to study the neutrophil response to infection in 2D environments.^[^ ^53^ ^]^ Human umbilical vein endothelial cells (HUVECs) were planked on porous membranes in the top well at 2 × 10^4^ cells per well overnight to form endothelial monolayers. The inflammation signals of *S. aureus* incubated with PBS, TTPy and RB were added to the bottom well. Neutrophils were isolated from whole blood of SD rat using a neutrophil isolation kit and then added into the top well at 5 × 10^5^ cells per well. The number of neutrophils that migrated across the endothelial layer into the bottom well at 1, 2, and 3 h of incubation was calculated.

### Immunohistochemistry Staining

MPO is the primary enzyme in a zurophilic granules of neutrophils. Immunohistochemistry analysis of MPO was used to observe the extravasate of neutrophils to the site of inflammation with the paraffin‐embedded section of vitreous body tissue at 1, 2, 3, 6, 12, and 24 h post‐treatment. Images were taken under an optical microscope.

### Statistical Analysis

All experiments data were presented as means ± standard deviation and performed in triplicate or more. Image J was used for quantification of inflammation cells and bacterial colonies. GraphPad Prism 8 and SPSS 23.0 software were used for statistical analyses. Unpaired two‐sided Student's *t*‐test was used for two‐group comparisons. One‐way ANOVA was performed among multiple groups. Statistically significant differences were represented with *P, ^#^ *P*, or ^*δ*^ *P* < 0.05, **P, ^##^ *P*, or ^*δδ*^ *P* < 0.01, and ***P, ^###^ *P*, or ^*δδδ*^ *P* < 0.001.

## Conflict of Interest

The authors declare no conflict of interest.

## Supporting information

Supporting Information

## Data Availability

The data that support the findings of this study are available from the corresponding author upon reasonable request.
