# Supplementary material for: Vision Defense: Efficient Antibacterial AIEgens Induced Early Immune Response for Bacterial Endophthalmitis
Source: Adv Sci (Weinh). 2022 Jul 6;9(25):2202485. doi: 10.1002/advs.202202485 (PMC9443450; doi:10.1002/advs.202202485)
Supplement: Supplementary file 1 — Supporting Information [file ADVS-9-2202485-s001.pdf]

ADVANCED SCIENCE
Open Access

Supporting Information

for *Adv. Sci.*, DOI 10.1002/advs.202202485

Vision Defense: Efficient Antibacterial AIEgens Induced Early Immune Response for Bacterial Endophthalmitis

Tingting Li, Yan Wu, Wenting Cai, Dong Wang, Chengda Ren, Tianyi Shen, Donghui Yu, Sujing Qiang, Chengyu Hu, Zheng Zhao, Jing Yu, Chen Peng* and Ben Zhong Tang**

Supporting Information

Vision Defense：Efficient Antibacterial AIEgens Induced Early Immune Response for Bacterial Endophthalmitis

Tingting Li, Yan Wu, Wenting Cai, Dong Wang, Chengda Ren, Tianyi Shen, Donghui Yu, Sujing Qiang, Chengyu Hu, Zheng Zhao, Jing Yu*, Chen Peng* and Ben Zhong Tang*

Supplementary Experimental Section

Materials

TTPy was synthesized and purified according to the previous report.[1] Other chemicals and solvents were bought from J&K Scientific and Sigma-Aldrich, and used as received. *S. aureus* was obtained from China General Microbiological Culture Collection Center. DMEM/F12 cell medium, penicillin, streptomycin and fetal bovine serum (FBS) were purchased from HyClone (USA). Cellular Reactive Oxygen Species Detection Assay Kitand Calcein/PI Cell Viability Assay kit were purchased from Beyotime (Shanghai, China). Cell counting kit (CCK)-8 was purchased from Yeasen (Shanghai, China). Bax and Bcl-2 antibodies were purchased from Proteintech (China). The ELISA kits were purchased from LIANKE(China). Anti-Myeloperoxidase antibody was purchased from Abcam (UK). Phosphate buffer saline (PBS) buffer (pH 7.2-7.4) and 0.9% normal saline were purchased from Procell (Wuhan, China). Distilled water was purified using a purification system (RSJ, China).

Characterization

TTPy powder was dissolved in dimethyl sulfoxide (DMSO) to obtain mother solution (5 mM), which was diluted with PBS for the following study. The OD value was measured using a multi-mode microplate reader (Molecular Devices SpectraMax iD5, USA). Photoluminescence (PL) spectra was collected on a fluorescence spectrometry (Agilent Cary Eclipse). The zeta potential of *S. aureus* was measured with a Malvern Zetasizer (ZEN3600 Nano ZS). The morphological changes of S. aureus were observed by a scanning electron microscopy (SEM, JEOL JSM-6390). Fluorescence images were collected on a fluorescence microscopy (Ti2-E, Nikon). Flow cytometric assays were performed on a flow cytometry (Becton Dickinson FACS Aria IIIu). Ultrasound pictures were obtained on small animal

ultrasound/photoacoustic imaging system (VEVO LAZR-X, VisualSonics).

Preparation of bacterial suspensions

A single colony on solid Luria broth (LB) culture medium for *S. aureus* was transferred to 5 mL of liquid culture medium and cultured shakily at 37 °C for 18 h. The concentrations of bacteria were determined by measuring the optical density at 600 nm (OD 600) and OD_{600} = 1.0 was considered as around 1×10^8 colony forming unit (CFU) mL^{-1}. Transferred to 1.5 mL centrifuge tube, bacteria were harvested by centrifugation at 9000 rpm for 3 min and washed with PBS for twice. The bacteria suspension was diluted to 1×10^6 CFU in a gradient for the following study. 100 μL bacteria dilution was added to TTPy at a certain concentration and 1 mL mixture system was obtained after dispersing with vortex. Prior to the irradiation, the mixture was incubated at room temperature for 15 min.

Hemolysis assay

Hemolysis assay was used to assess the hemocompatibility of TTPy. Whole blood was washed several times with saline until the supernatant became colorless. Diluted with saline to 2% red cell suspension, the obtained erythrocytes were incubated with different TTPy concentrations (0, 0.02, 0.05, 0.1 and 0.2 μM) for 60 min at 37 °C. Then the solutions were centrifuged (3000 rpm, 5 min). The photos were taken and the OD value of supernatants at 540 nm was recorded by a microplate reader. The ratio of hemolysis was calculated according to the received formula. The cell suspension added with distilled water was regarded as positive control.

Staining of frozen section of eyeball

S. aureus was collected according to the above steps and injected to the vitreous cavity of rat eyes. Subsequently, 10 μL TTPy and RB at 0.1 μM were injected respectively after *S. aureus*. Then the eyeballs were removed after incubation with TTPy and RB for 15 min. The frozen sections of eyeballs were obtained on a frozen section machine. Pictures were taken on a fluorescence microscope (Leica, Germany).

Supplementary figures

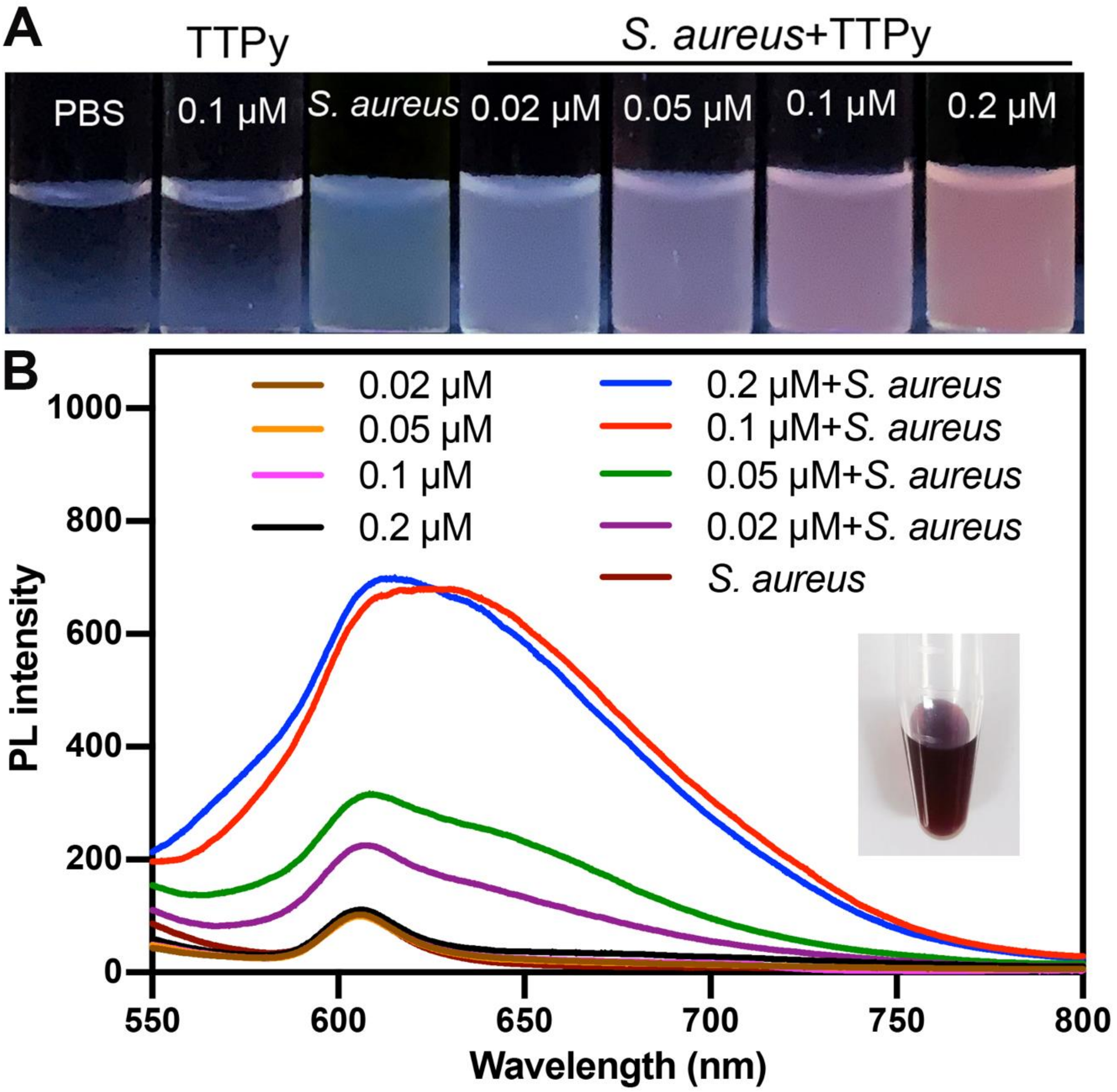

Figure S1. A) The picture of *S. aureus* incubated with TTPy at different concentrations for 15 min under 365 nm UV light. B) PL spectra of TTPy with or without incubation with *S. aureus* for 15 min (Excitation wavelength = 489 nm). The insert was the photograph of TTPy solution (5 mM) dissolved in DMSO.

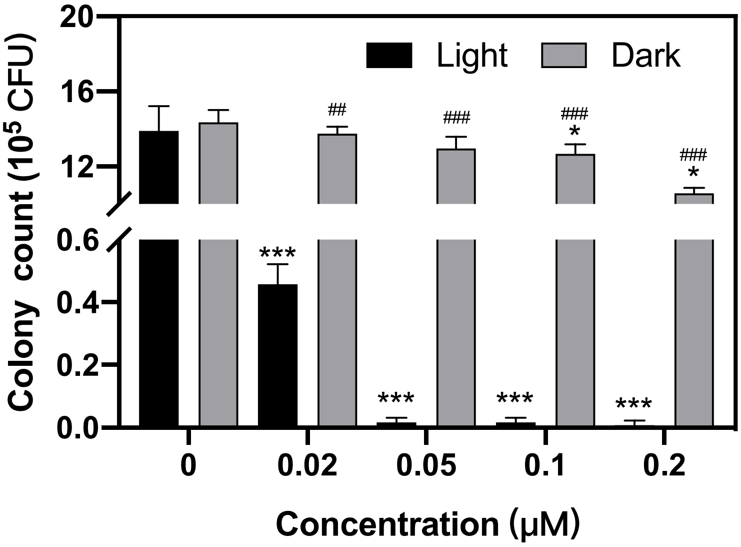

Figure S2. The surviving colony count of *S. aureus* treated by TTPy in light condition (20 mW cm^{-2}) for 10 min (* for comparison among different concentrations of TTPy (0.02, 0.05, 0.1, 0.2 μM) *versus* TTPy (0 μM) in light and dark conditions, # for comparison between light and dark at the same concentration. * P or # P < 0.05, ** P or ## P < 0.01, and *** P or ### P < 0.001).

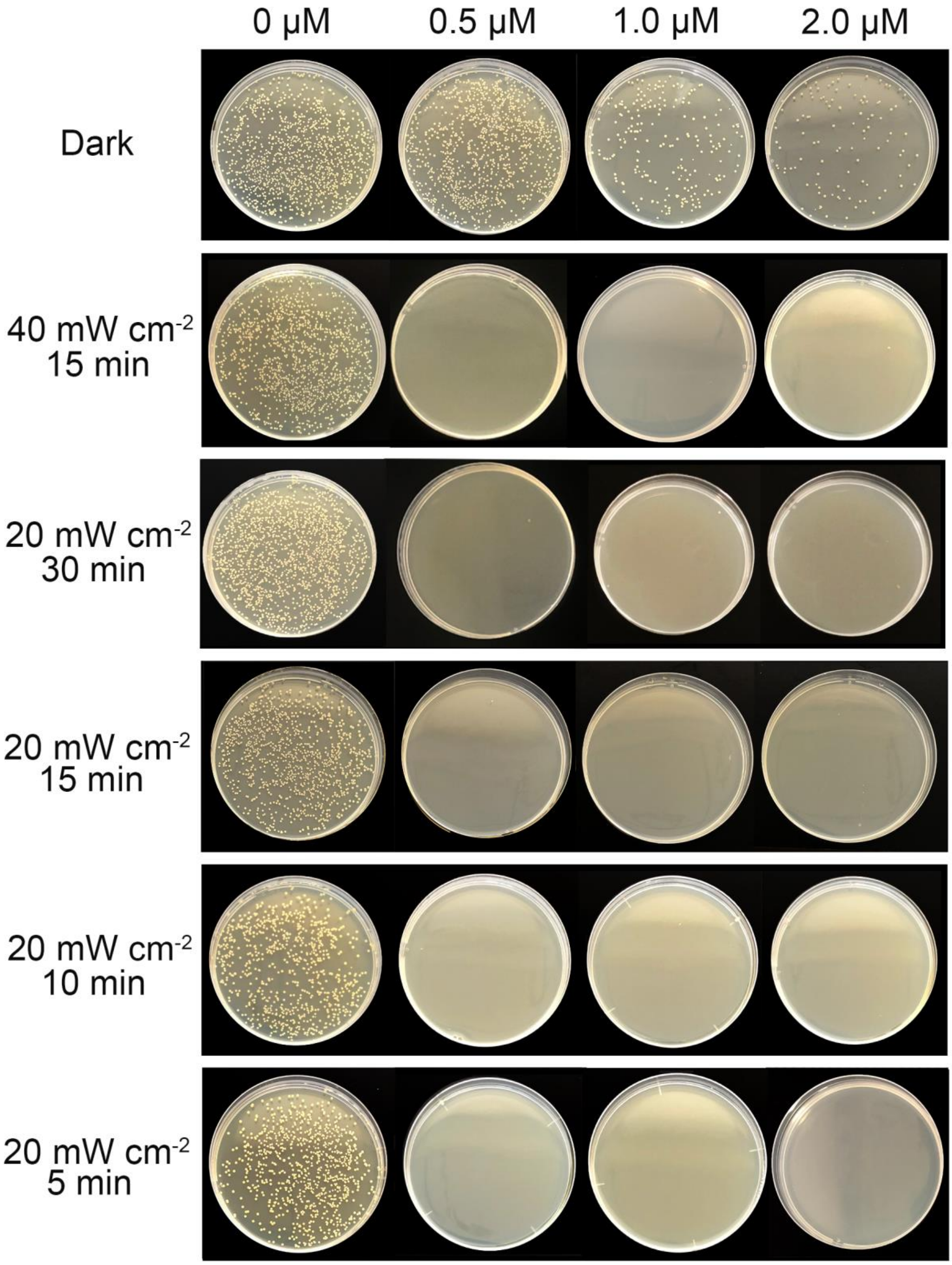

Figure S3. Representative photographs of *S. aureus* treated by TTPy with a concentration range of 0.5-2.0 μM under different irradiation conditions.

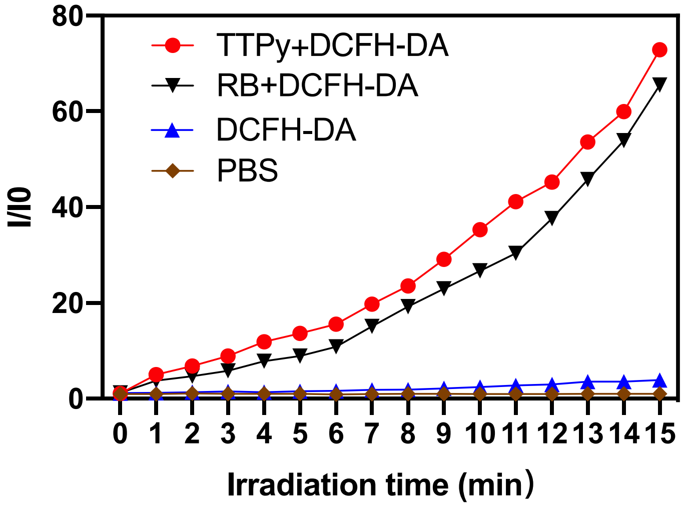

Figure S4. ROS generation of TTPy and RB at 0.1 μM under light irradiation (20 mW cm^{-2}) for 15 min.

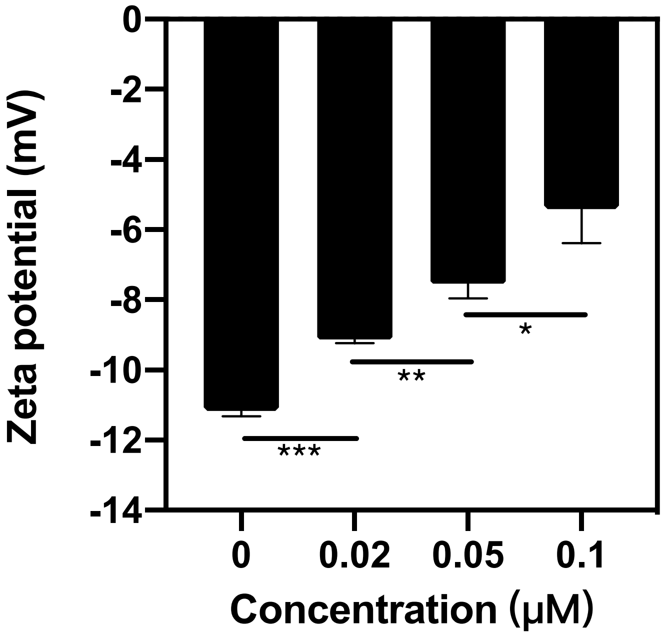

Figure S5. Zeta potentials of *S. aureus* incubated with different concentrations of TTPy for 15 min (* P < 0.05, ** P < 0.01, and *** P < 0.001).

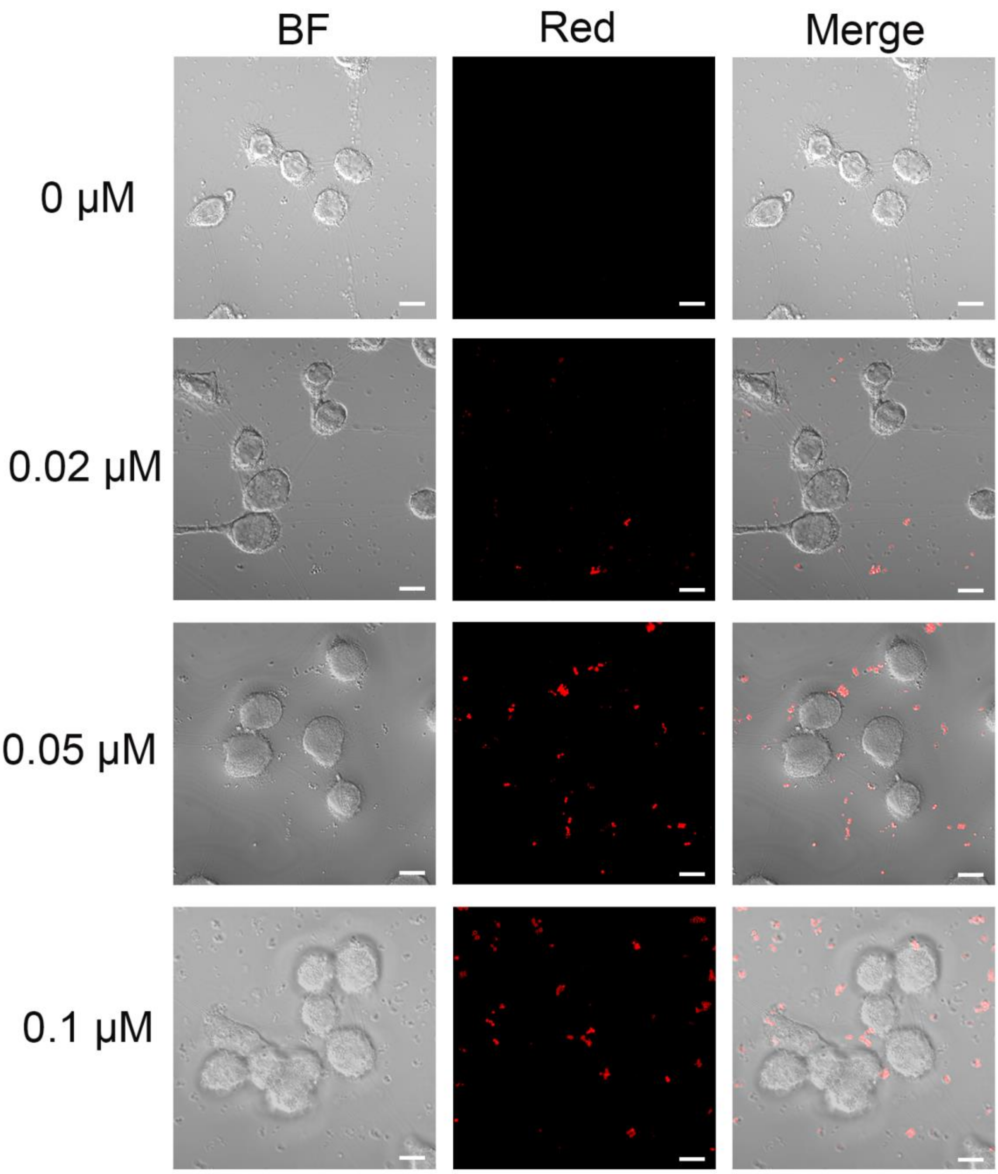

Figure S6. Representative fluorescence images of *S. aureus* and ARPE-19 cells co-cultured with TTPy for 15 min (Excitation wavelength = 488 nm, scale bar: 10 μm).

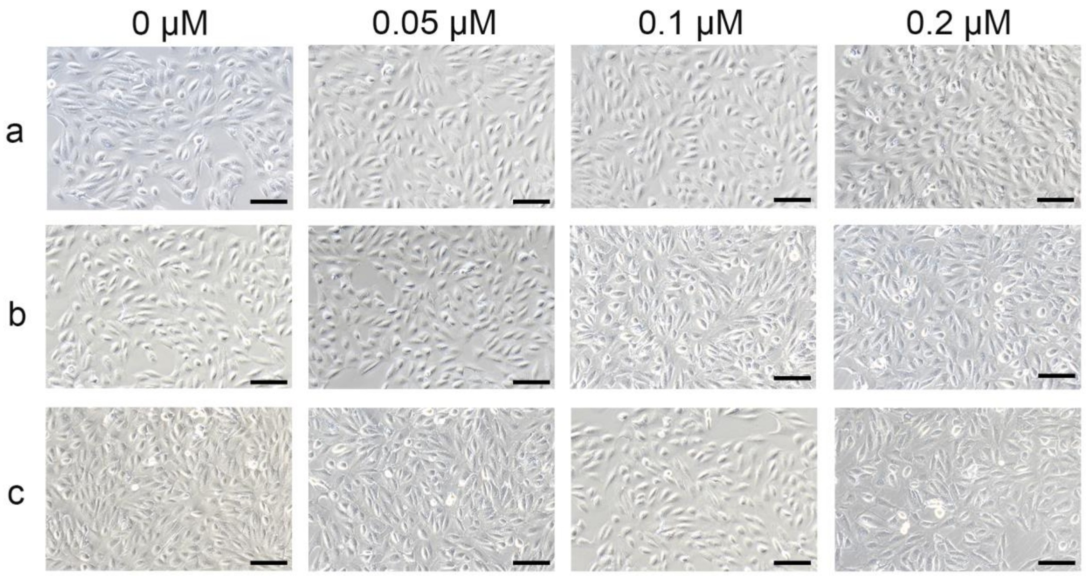

Figure S7. Cell morphology of ARPE-19 cells incubated with different concentrations of TTPy in different irradiation conditions. (a and b) treated for 15 min a) with light and b) without light after incubation with TTPy for 15 min, c) treated by TTPy for 24 h without light (scale bar: 100 μm).

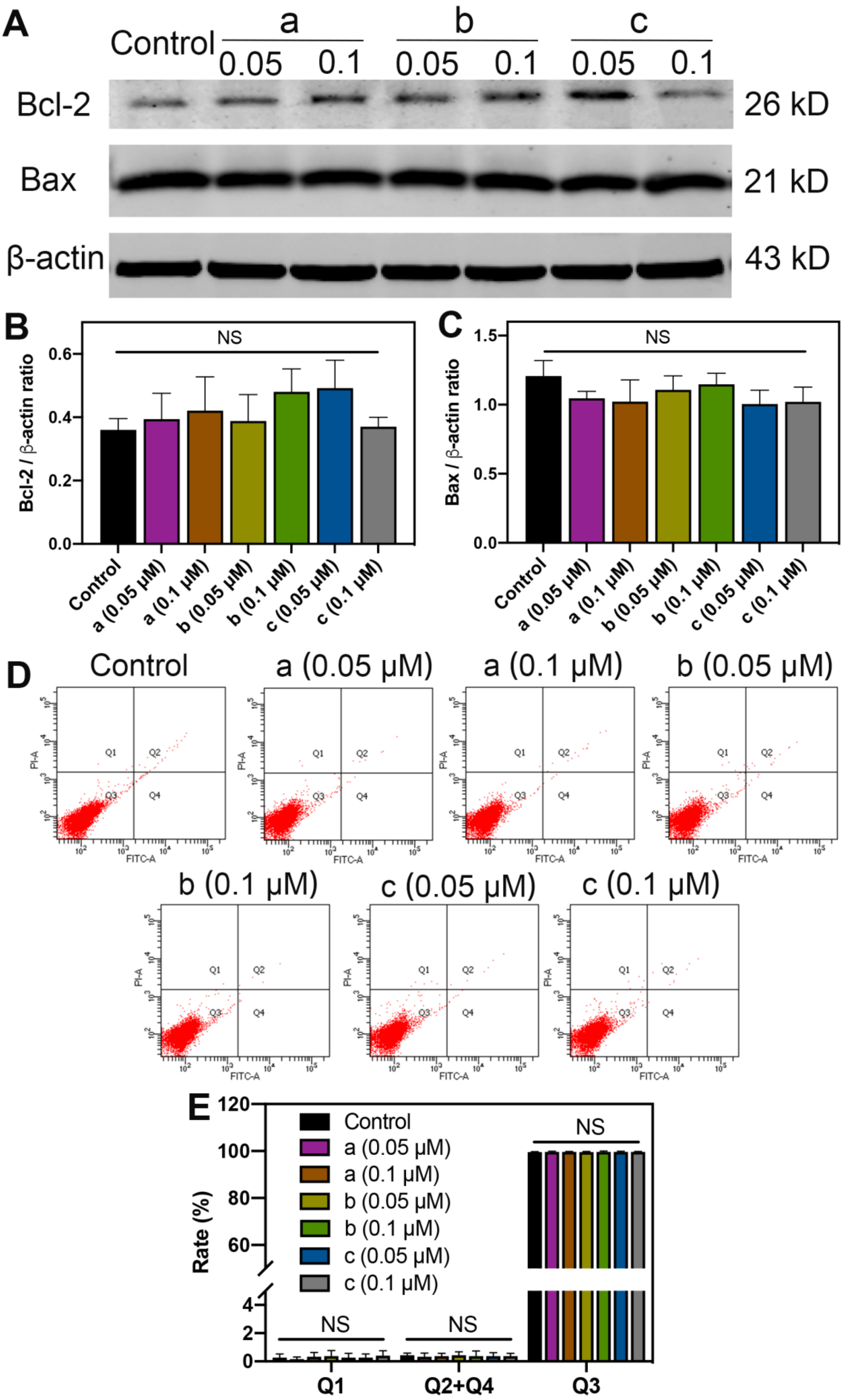
A
Control
a
b
c
0.05 0.1 0.05 0.1 0.05 0.1
Bcl-2
26 kD
Bax
21 kD
β-actin
43 kD
B
Bcl-2 / β-actin ratio
NS
C
Bax / β-actin ratio
NS
Control
a (0.05 μM)
a (0.1 μM)
b (0.05 μM)
b (0.1 μM)
c (0.05 μM)
c (0.1 μM)
D
Control
a (0.05 μM)
a (0.1 μM)
b (0.05 μM)
b (0.1 μM)
c (0.05 μM)
c (0.1 μM)
PI-A
FITC-A
Q1
Q2
Q3
Q4
E
Rate (%)
Control
a (0.05 μM)
a (0.1 μM)
b (0.05 μM)
b (0.1 μM)
c (0.05 μM)
c (0.1 μM)
NS
Q1
Q2+Q4
Q3

Figure S8. A) Western blot of ARPE-19 cells to detect the expression of apoptosis protein. (B and C) The ratio of B) Bcl-2/β-actin and C) Bax/β-actin. D) Flow cytometry of ARPE-19 cells incubated with TTPy under different irradiation conditions by different fluorescent light channels. E) The statistical analysis of ARPE-19 cells in Q1, Q2+Q4 and Q3. (a and b) treated for 15 min a) with light and b) without light after incubation with TTPy for 15 min, c) treated by TTPy for 24 h without light.

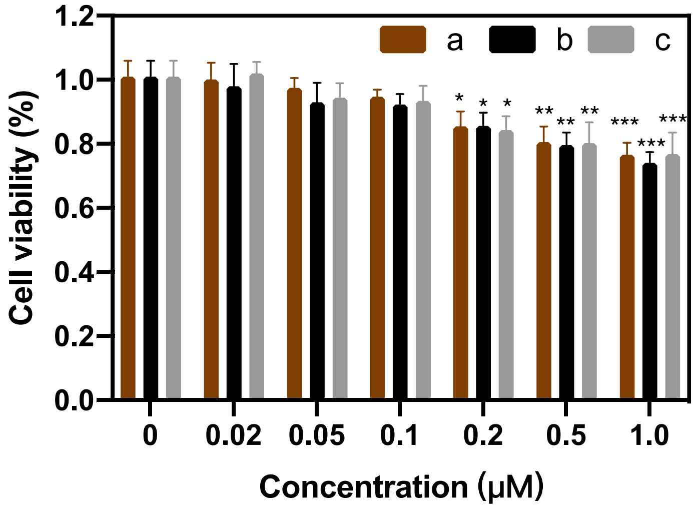

Figure S9. Cell viability of HCE-T cells treated by TTPy at different concentrations and irradiation conditions. (a and b) treated for 15 min a) with light and b) without light after incubation with TTPy for 15 min, c) treated by TTPy for 24 h without light (* for comparison among different concentrations of TTPy (0.02, 0.05, 0.1, 0.2, 0.5 or 1.0 μM) *versus* TTPy (0 μM) in group a, b and c, # for comparison between group a and b, and δ for comparison between group a and c under the same concentration. NS, not significant, * $P < 0.05$, ** $P < 0.01$ and *** $P < 0.001$).

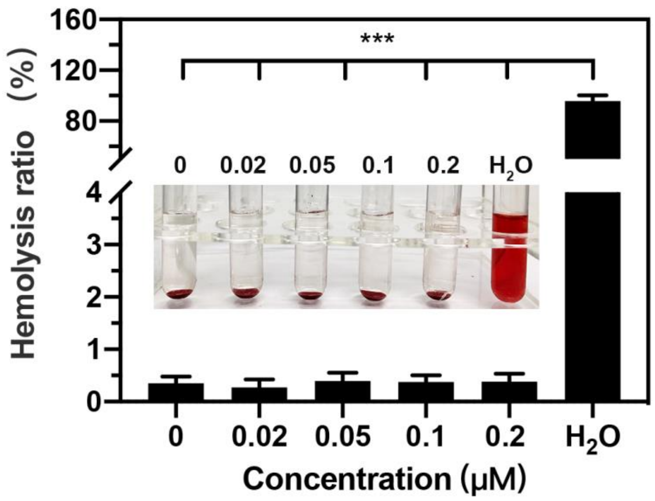

Figure S10. Hemolysis assay of RBCs treated by TTPy at different concentrations (* for comparison among different concentrations of TTPy (0, 0.02, 0.05, 0.1 or 0.2 μM) *versus* distilled water, *** $P < 0.001$).

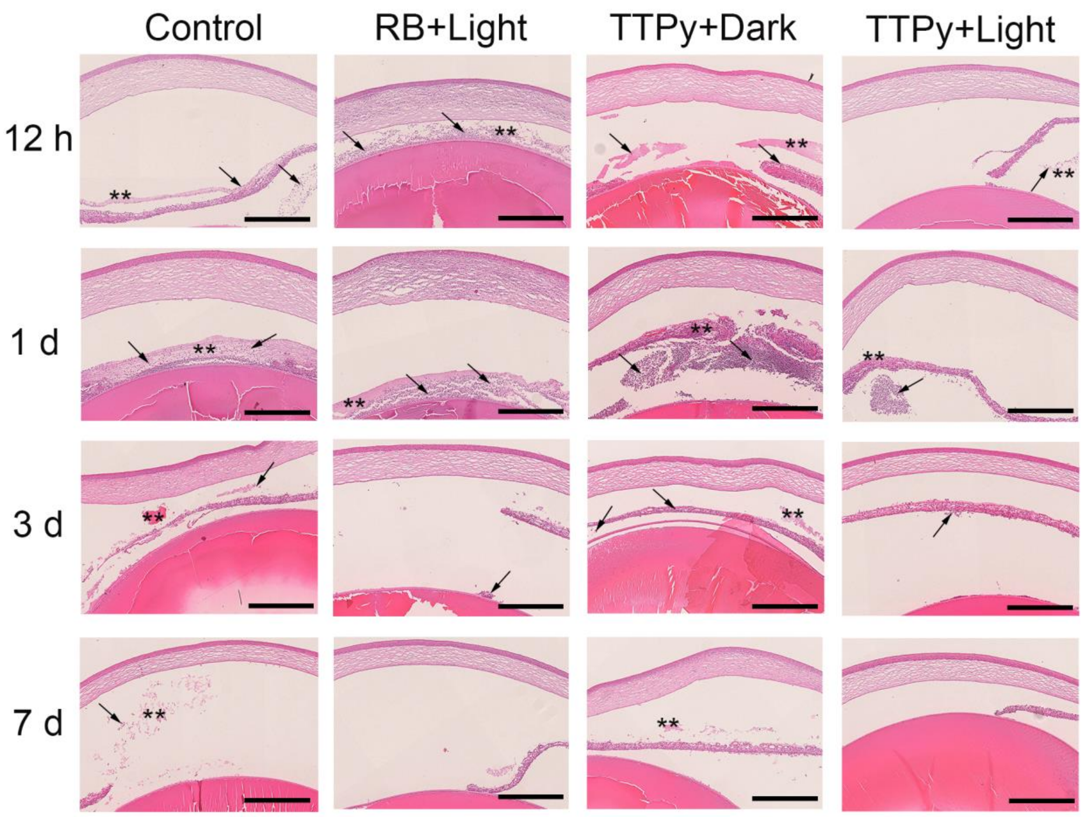

Figure S11. H&E staining of anterior chamber at different time points after different treatments. ** fibrinous exudate, black arrows: inflammatory cells (scale bar: 500 μm).

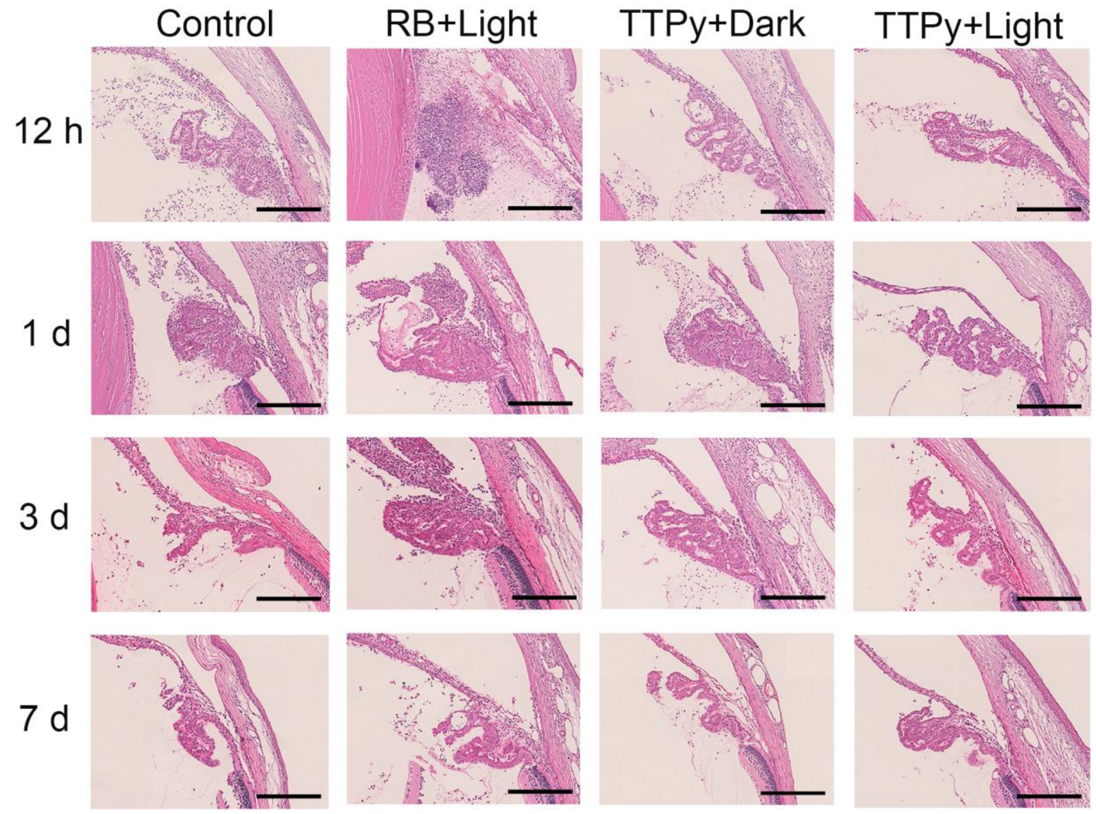

Figure S12. H&E staining of ciliary body at different time points after different treatments (scale bar: 250 μm).

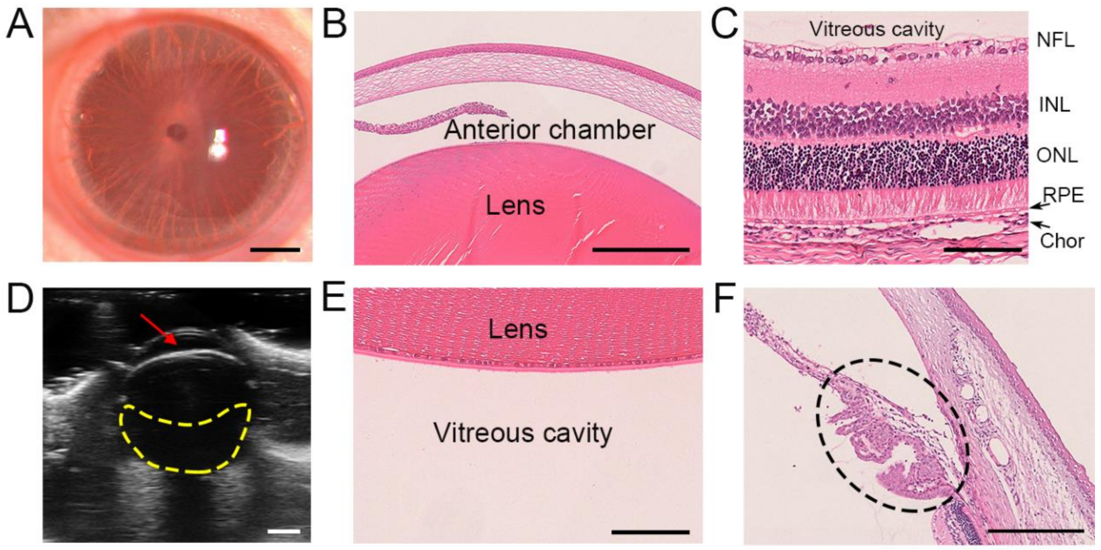

Figure S13. Normal structure of the rat eyeball. A) The ocular surface of rat eyeball with transparent cornea, normal iris vessels and pupil (scale bar: 1mm). (B, C, E and F) Representative H&E images of B) clear anterior chamber between cornea and lens (scale bar:

500 μm), C) retina architecture with different layers (scale bar: 100 μm), E) vitreous cavity (scale bar: 100 μm), and F) ciliary body (scale bar: 250 μm). D) Ultrasound image of whole eyeball with anterior chamber (red arrow) and vitreous cavity (yellow dotted lines) (scale bar: 500 μm).

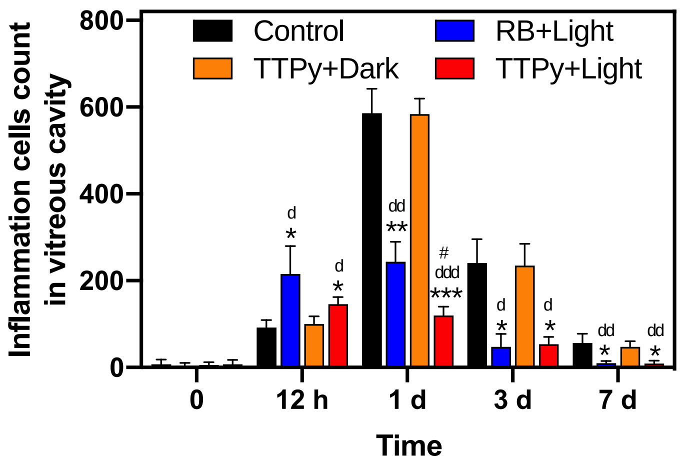

Figure S14. The inflammatory cells count in vitreous cavity at different time points after treatment (* for group RB+Light, TTPy+Dark, TTPy+Light versus group Control at the same time point, # for group RB+Light versus group TTPy+Light, δ for group RB+Light, TTPy+Light versus group TTPy+Dark. * P, # P or δ P < 0.05, ** P, ## P or δδ P < 0.01, and *** P, ### P or δδδ P < 0.001).

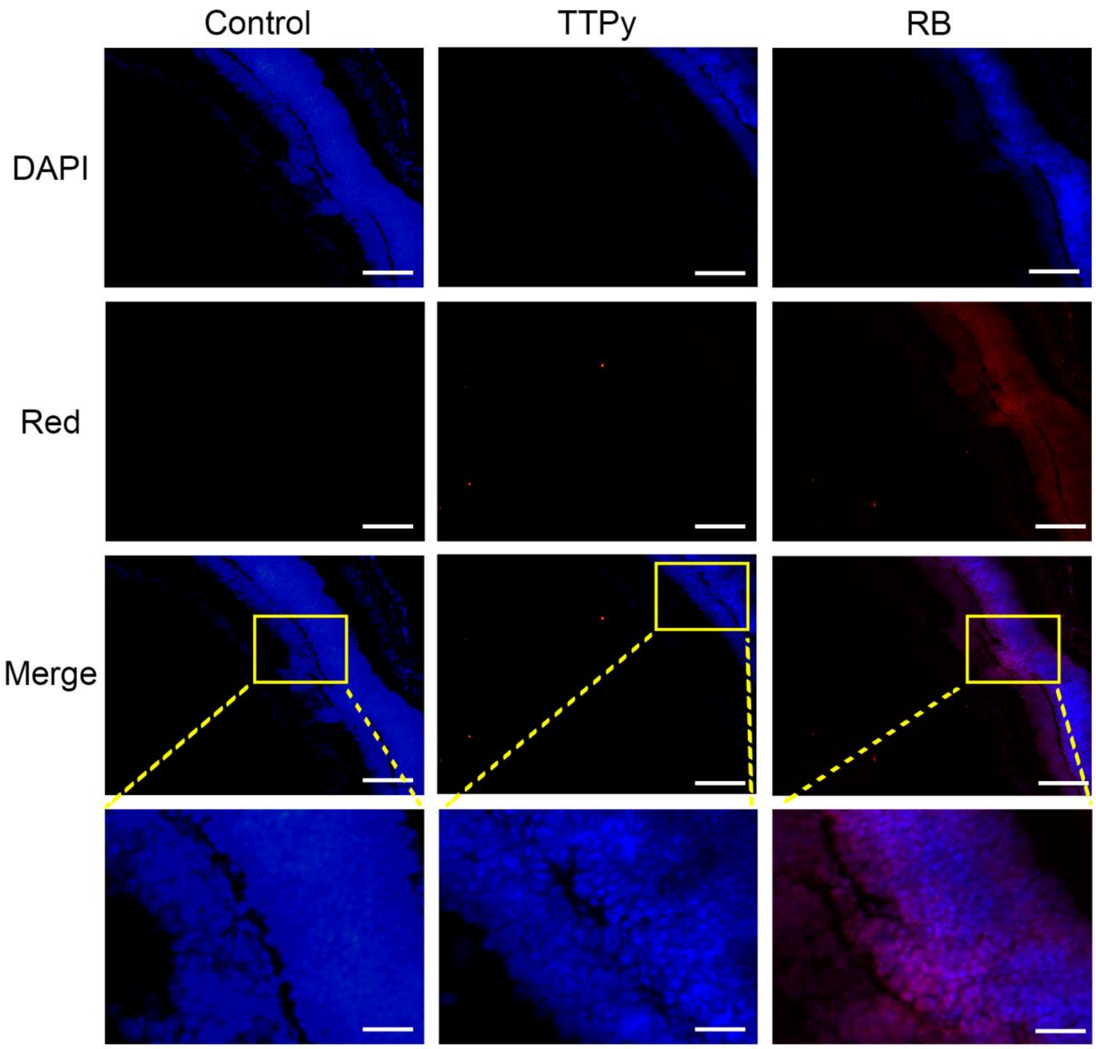

Figure S15. Fluorescence images of frozen section of eyeball after intravitreal injection of *S. aureus* incubated with TTPy or RB for 15 min (scale bar: 100 μm for original images, 20 μm for amplified images).

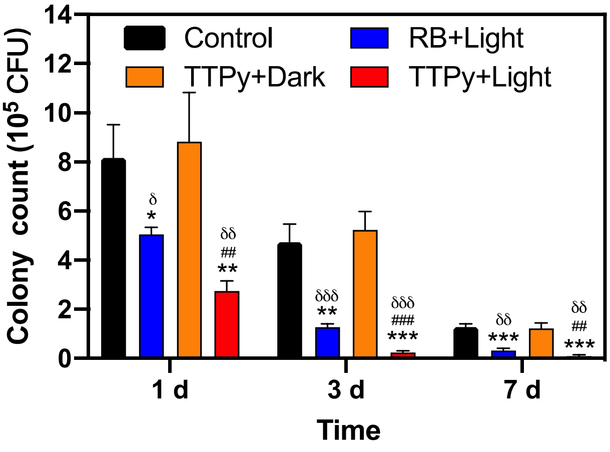

Figure S16. The surviving colony count of *S. aureus* in vitreous cavity after different treatment at different time points (* for group RB+Light, TTPy+Dark, TTPy+Light versus group Control at the same time point, # for group RB+Light versus group TTPy+Light, δ for group RB+Light, TTPy+Light versus group TTPy+Dark).

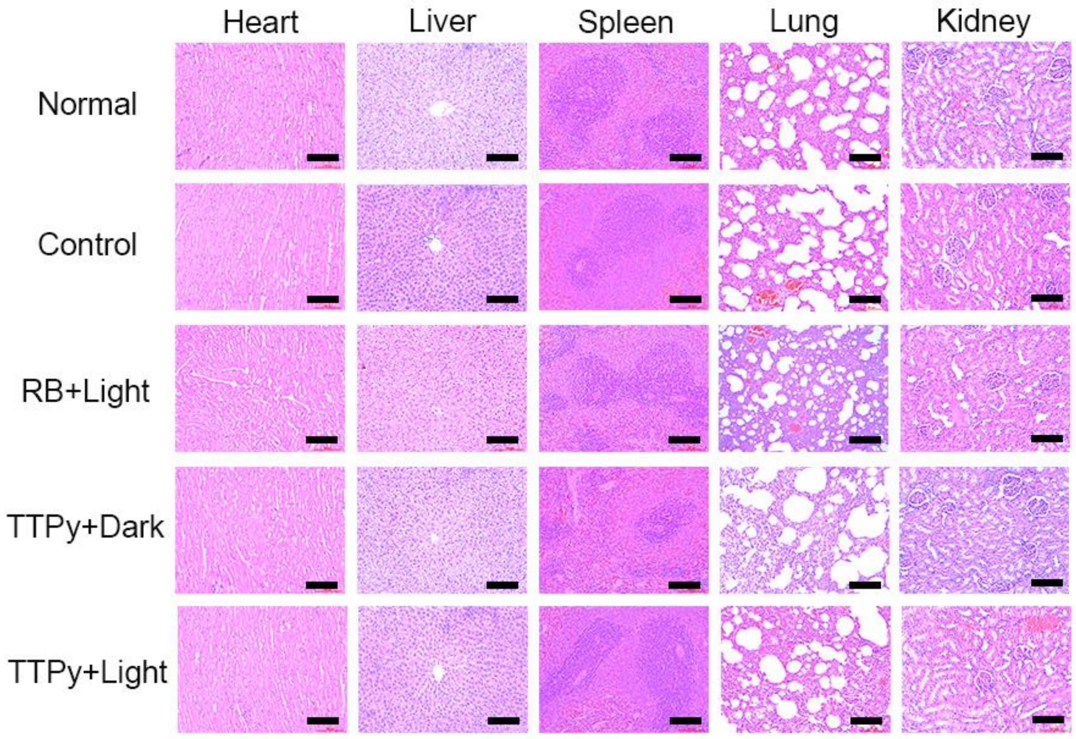

Figure S17. H&E staining of main visceral organs at 7 d post treatment (heart, liver, spleen, lung and kidney, scale bar: 100 μm).

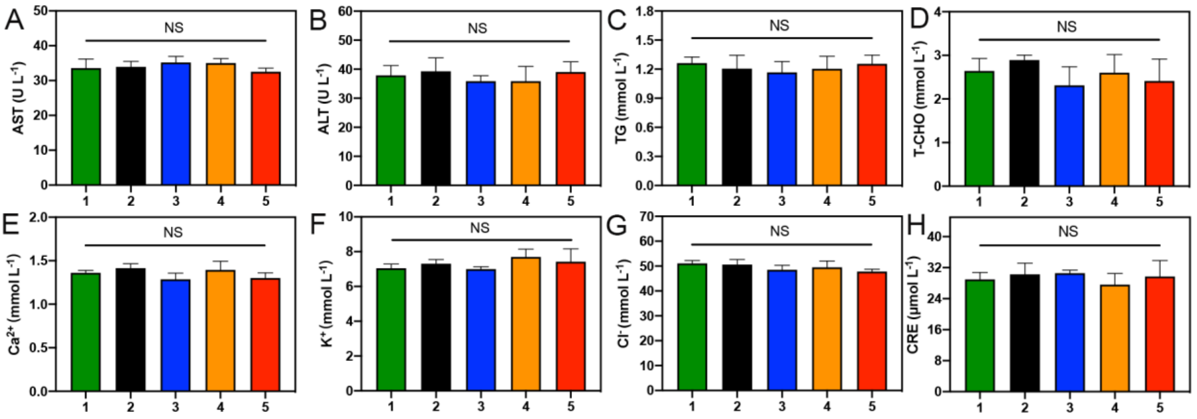

Figure S18. Blood biochemistry examination analysis of rats after different treatments. A) AST B) ALT C) TG D) T-CHO E) Ca^{2+} F) K^+ G) Cl^- H) CRE. Group Normal (1), Group Control (2), Group RB+Light (3), Group TTPy+Dark (4) and Group TTPy+Light (5) (NS, not significant).

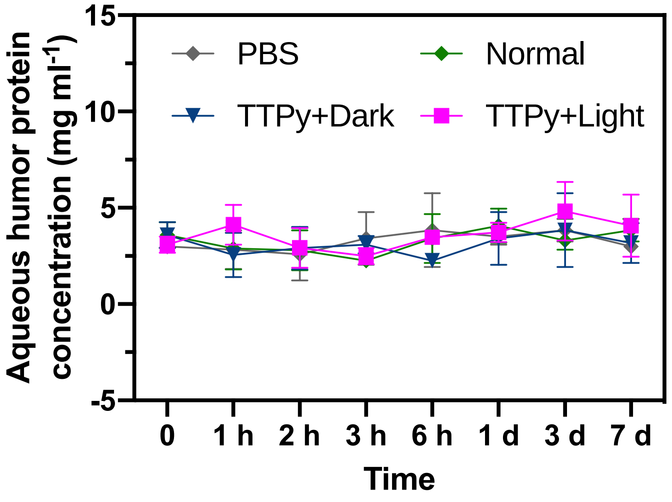

Figure S19. The concentration of aqueous humor protein after intravitreal injection of PBS and pure TTPy in light and dark conditions.

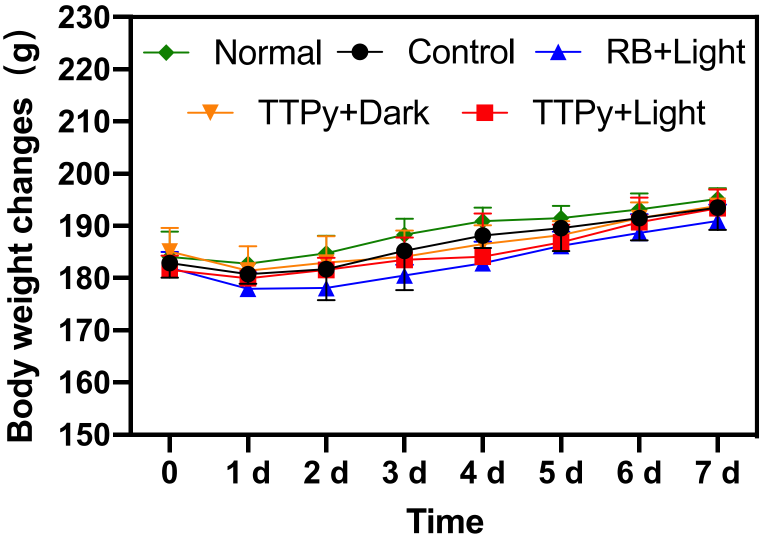

Figure S20. Changes of body weight in the observation course of 7 days.

References

[1] D. Wang, M. M. S. Lee, G. Shan, R. T. K. Kwok, J. W. Y. Lam, H. Su, Y. Cai, B. Z. Tang, *Adv Mater.* **2018,** *30*, 1802105.
